# Autonomous Continuation of Community Health Workers' Activities in Thegon Township, Bago Region, Myanmar

**DOI:** 10.3389/fpubh.2020.00201

**Published:** 2020-06-02

**Authors:** Taeko Oguma, Etsuko Watanabe, Tomoari Mori, Yasuyuki Fujino

**Affiliations:** ^1^Takemi Program in International Health, Harvard T. H. Chan School of Public Health, Boston, MA, United States; ^2^Department of Public Health, Graduate School of Medicine, University of Tokyo, Tokyo, Japan; ^3^Division of Nursing, Faculty of Healthcare, Tokyo Healthcare University, Tokyo, Japan; ^4^Office of the Dean of Research, Okinawa Institute of Science and Technology Graduate University, Okinawa, Japan; ^5^People's Hope Japan, Tokyo, Japan

**Keywords:** activity continuity, sustainability, autonomy, community health worker, empowerment, low-middle income country, supervision

## Abstract

**Background:** Few studies have reported on the autonomous continuation of Community Health Worker (CHW) activities after external supervision and support have ended.

**Objective:** The study reports CHW activity continuation in Thegon Township, Bago Region, Myanmar, observed after the supervision by the external organization is completed.

**Method:** Following the completion of a child nutrition program in Thegon Township, CHWs were left unsupervised and uninformed of a follow-up at 10, 23, and 40 months from the end of the program survey due to unforeseen circumstances. In a follow-up survey in 2014, data on the activity implementation status from CHWs and activity attendance from caregivers of the target children were collected. Focus group discussions were held with caregivers concerning their information sources on child nutrition and health.

**Results:** On average, CHWs were found to have continued with 2.6 of the four core activities, often with modifications, irrespective of the time since completion of the non-profit-organization-led program. Meanwhile, caregiver attendance decreased over time. Caregivers recognized CHWs as information sources.

**Discussion:** Although unsupervised, CHWs ambitiously continued with their activities, but sorted through and modified them, which may have been unrelated to the local acceptance of the program, as caregiver attendance decreased even as CHWs continued the activities. The observation may highlight the importance of proactive engagement and thus, the autonomy of CHWs in their activity continuation.

## Introduction

Increasing attention is being paid to public health program sustainability, although few programs have evaluated their long-term impacts beyond their active engagement (1–4) as a result of methodological constraints, including uncertainty in relation to terminology (3, 5).

Pluye summarized that program sustainability matters because the programs' long-term effects are indispensable in relation to individuals' behavioral changes, and thus their ultimate health outcomes (6). Program sustainability is also essential in relation to avoiding “investment losses” and “disillusionment” among the participants, which could make subsequent community intervention difficult (6).

Moore synthesized a concept of program sustainability, taking into account the differences in sustainability between the individual and program or system levels (3). Two child nutrition intervention programs found their temporal individual-level health effects possibly only while the recipients were under the programs (7, 8), whereas a family planning program reported its long-lasting impact after funding ended, on the recipients' service utilization as well as organizational system-level activity continuation (9).

In low-middle income contexts, the programs can be less sustainable due to financial, infrastructural, and system obstacles (1, 4). However, utilization of limited but existing resources and program flexibility are factors that promote program sustainability (4), and partial sustainability was more common than the continuation of the activities that were initially introduced (2).

Community health workers (CHWs) play various roles in maternal and child health care, especially in middle- and low-income countries (10–16). Various non-monetary and monetary incentives have been described as ways to ensure good CHW performance (10, 15, 17, 18).

While quality supervision is one incentive that has been found to motivate CHWs (10, 18, 19), years of supervision may be unrealistic (20) for aid activities that are limited in terms of budget, time, and human resources; furthermore, data about CHW supervision is sparse (18, 19, 21).

Ozano reported that CHWs in Cambodia had their own ideas about overcoming resource restrictions by modifying their assignments, as well as the skills to do so (22). Kane mentioned that supervisions overfocusing on data collection and reporting and fault-finding discouraged CHWs, and discussed the importance of CHWs' empowerment themselves and their control over their own work (23). Thus proactive, autonomous (23) engagement of CHWs in their activities is an essential factor in program sustainability, yet little is known about the CHWs' self-sustained activities without supervision and support from donor organizations.

The study describes an activity continuation of the CHW in Thegon Township, Bago Region, Myanmar, observed after the completion of a child nutrition improvement program implemented by a non-profit organization (NPO).

## Method

The NPO (Save the Children) conducted a survey months after the Child Nutrition program comprising Infant and Young Child Feeding (IYCF) (24) and livelihood supporting had completed, in Thegon Township, Bago Region, Myanmar. The NPO reported the results of the survey to the Japanese Ministry of Foreign Affairs in 2015. This is a secondary analysis of the data obtained by the NPO.

### Local Setting

Thegon Township was a rural agricultural area, and residents were primarily peasant farmers living in poverty. Thirty-four percent of children aged <60 months were stunted, with 8% demonstrating acute wasting (Save the Children, 2007, unpublished). These numbers are consistent with trends in other areas of the country where 35% of children were stunted and 7.9% wasting in 2009–2010 (25).

### NPO-led Program and CHW Training

The NPO had implemented a series of year-long nutrition and livelihood supporting intervention programs in a total of 90 villages divided into three groups (group 1 in 2010, 2 in 2011, and 3 in 2012), targeting under 60 months children and their mothers, pregnant women. The program aimed to expand the knowledge and practice of IYCF (24) among the caregivers of the young children. Livelihood support activities such as home gardening, chicken rearing, bicycle-share groups, and saving activity (Group 1 and 2) were also introduced.

Lay, unpaid CHWs specialized in child nutrition-related activities were selected and trained. In each village, the core village members including the village chief, religious leader, and midwives working in the village nominated three male or female CHWs to address nutrition-related activities as “nutrition volunteers,” regardless of the size of the village. The core village members also chose three female CHWs to provide counseling in relation to breastfeeding as “peer counselors.” The NPO designed five core CHW activities based on Growth Monitoring and Promotion (GMP) (20, 26) as follows:

- “Growth Monitoring” (measure child's weight/height and compare these with growth standards; report undernourished cases to local midwives): conducted monthly in Group 1 villages, quarterly in other villages, or monthly for undernourished cases
- “Nutrition Education” seminar for caregivers: conducted monthly
- “Peer Counseling” for breastfeeding caregivers: conducted monthly; Peer Counseling was introduced after the program ended for Group 1
- “Cooking Demonstration” for caregivers: conducted monthly in Group 1, and twice a year in Groups 2 and 3
- “Home Visit” for undernourished children: conducted monthly

The NPO offered initial training for CHWs for 4 days in Group 1, and 3 days in Group 2 and 3, wherein the midwife from the local health center and the local NPO staff members lectured CHWs on how to implement each activity. During the NPO-led program periods, the CHWs reported to the local midwives each month regarding their activities, such as the topics covered in their nutrition education talks, the numbers of participants, the frequency of the talks, and the results of their home visits. The local midwives provided feedback to the CHWs on the appropriateness of their talk topics and teaching materials.

After the completion of the NPO-led program for Group 3, NPO supervision (monitoring and meetings) and refresher training for all CHWs ended. Information on the quality of supervision, the supervisors' training, the refresher training, and CHW retention or replacement during and after the programs is unavailable.

### Follow-Up Survey

Ten, 23, and 40 months after the program ended in 2014, the NPO took advantage of an opportunity to conduct follow-up surveys in all 90 villages aimed at evaluating the lasting effects of the program. The NPO evaluated the situation regarding CHW activities and caregivers' attendance at those activities. The role of the CHWs as an information source for caregivers was also explored through focus group discussions. CHWs were not made aware of the surveys in advance.

#### CHW Activity Continuation

Face-to-face questionnaire survey was conducted by local NPO staff wherein CHWs in all 90 villages were questioned on the forms and frequency (27) of the typical five activities. Any of the CHWs from each village were able to respond after providing written consent. The questionnaire asked the CHWs to choose one of the following options regarding the form of each activity:

- Done exactly the same way as it was introduced
- Done with modification
- Totally abandoned.

They were also asked to choose one of the following options in relation to frequency:

- Every month
- Every other month
- Less than five times per year
- Zero
- Only once undernourished child is identified (only in case of Home Visit).

#### Caregiver Attendance at CHW Activities

A face-to-face questionnaire survey was also conducted in all 90 villages by local NPO staff wherein caregivers were questioned on their IYCF-related knowledge and practices and their prior attendance (“Never attended” or “Attended”) at IYCF-related CHW activities. All of the primary caregivers with children aged under 24 months, as well as 40% of those primary caregivers with children aged 24–59 months who were randomly selected based on the number of children of the same age group registered in each village were invited to participate in the questionnaire survey. Considering the local cultural context, female caregivers were regarded as the primary caregiver regardless of whether they were the children's mothers, and verbal consent was obtained prior to the surveys.

#### Focus Group Discussion (FGD) With Caregivers and Pregnant Women

One-to-two-hour Focus Group Discussions (FGD) were held in public places in six randomly selected villages (two from each Group). The village CHWs invited five or six caregivers with children aged <60 months or pregnant women to participate in the focus groups and discuss their information sources regarding IYCF and child health. Written consent was obtained prior to data collection, including recording. The ages of the participants, number and ages of children, and months of gestation in the case of pregnant women were obtained prior to the discussions.

The discussions were facilitated by a Burmese medical doctor who was a staff member of the NPO, accompanied by another staff member who recorded proceedings, and two note-takers from the local NPO office. The conversations were recorded to help the note-takers fill in any parts of the conversations they had missed and to confirm their understanding of what was said. The discussion topic was “How information on baby/child care is transferred,” and the facilitator paraphrased their questions in an arbitrary manner to stimulate conversation, based on the following concepts:

- How does the information cascade between generations?
- Is there communication with other experienced mothers?
- Who provides information to the caregivers?
- How do the caregivers obtain information?

After the facilitator translated the original notes from Burmese into English, two non-Burmese NPO staff members, the facilitator, and the two note-takers reviewed the meaning of each response after considering the local context.

### Analysis

The CHW activities were classified as “Same” if they had not changed, “Discontinued” if they had been abandoned, and “Modified” in all other cases. We define that an activity was considered to have been “maintained” unless the CHW advised that the activity had been totally abandoned, and the CHW “activity continuation” as the number of conducted activities (with the form described as “Same” or “Modified”) for all five activities except for the Home Visit, which designed solely for undernourished children. We divided the villages into two groups according to their level of CHW “activity continuation” (≤2 or ≥3) based on the numbers of activity continuation and calculated the elapsed time from the end of the program.

Time-dependent trends in CHW activity continuation (≤2 or ≥3) and caregiver attendance (“Never Attended” or “Attended”) were examined using the Cochran–Armitage test. The notes from the FGDs were reviewed and categorized according to information sources.

## Results

**Figure 1:**
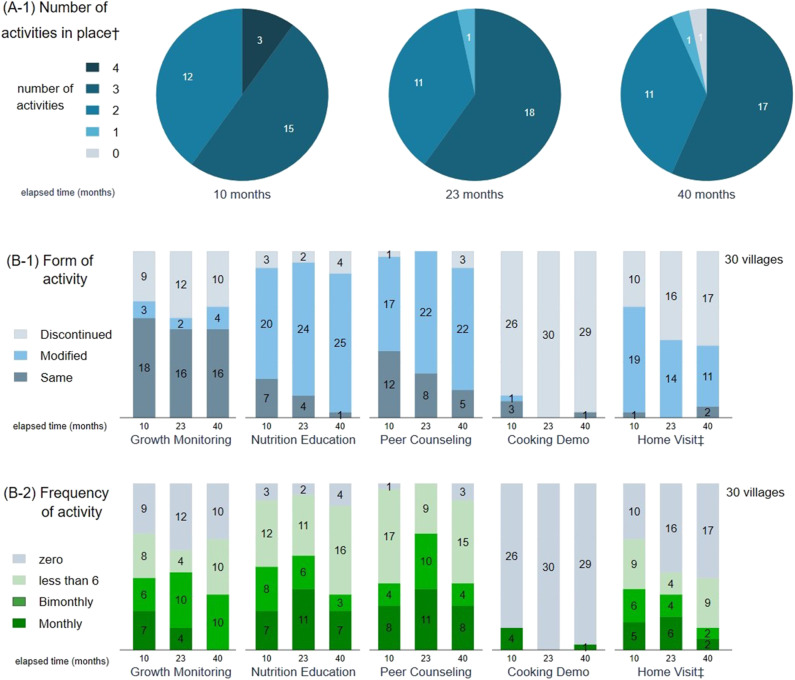
Continuation of Community Health Workers' activities in place among 30 villages by elapsed time: Numbers, Form, and Frequencies. ^†^One response regarding Nutrition Education was considered “Discontinued” as it was unclear. ^‡^Home visit: solely for undernourished children. **(A-1)** The mean number of continued activities was 2.6 among the four core activities (Growth Monitoring, Nutrition Education, Peer Counseling, and Cooking Demonstration). **(B-1)** Activity continuation rates were 65.6% for Growth Monitoring, 90.0% for Nutrition Education, 95.6% for Peer Counseling, and 5.6% for Cooking Demonstration. Activity form modification occurred independently of the elapsed time but was dependent on each activity. **(B-2)** Activity frequencies varied.

Responses were obtained from CHWs in all 90 villages. Most villages had been able to maintain at least two activities, although one village had discontinued all activities (Figure 1A-1). Among the four core activities, the mean number of continued activities was 2.6. Activity continuation rates were 65.6% (59/90) for Growth Monitoring, 90.0% (81/90) for Nutrition Education, 95.6% (86/90) for Peer Counseling, and 5.6% (5/90) for Cooking Demonstration. Activity forms were frequently modified; this occurred independently of the elapsed time but was dependent on each activity (Figure 1B-1). Activity frequencies also varied (Figure 1B-2), and there was no significant elapsed time-dependent trend in activity continuation (Table 1).

**Table 1:** Community Health Worker activity continuation and caregiver attendance at any of the activities, according to time elapsed from NPO-led program completion.

**Elapsed time (months)**	Villages with activity continuation (%)^*^		Caregiver attendance (%)^**^	
	**≤2**	**3≤**	**Never attended**	**Attended**
10	12 (40.0)	18 (60.0)	46 (14.4)	274 (85.6)
23	12 (40.0)	18 (60.0)	94 (30.5)	214 (69.5)
40	13 (43.3)	17 (56.7)	186 (45.0)	227 (55.0)
Total	37 (41.1)	53 (58.9)	326 (31.3)	715 (68.7)

* *One response regarding Nutrition Education was considered “Discontinued” as it was unclear*.

** *p for trend <0.05*.

A median of 14 (range, 2–47) primary caregivers from each village responded, with an overall response rate of 90.9%. The majority (68.7%, 715/1,041) responded that they had attended CHW activities by the time of the survey, but attendance rates decreased significantly with time (Table 1).

In the focus groups, a total of 35 caregivers or pregnant women, with a median age of 30 (range 18–44) years old, and a median child number of 1 (range 0–5), participated. Ten of them were pregnant, and seven were in their first pregnancies. Although, in all six villages, expansion of the discussion by the participants themselves was extremely difficult, they provided their various information sources, as follows:

- CHW activity: Growth Monitoring, Nutrition Education, and Cooking Demonstration.
- Other sources: campaign events by other organizations, health care professionals (NPO staff/midwives/doctors/other hospital staff), casual conversation (i.e., chatting with other villagers), and media (magazines/radio).

## Discussion

In the surveyed villages, CHWs had maintained their activities for up to 40 months after the end of the NPO-led program. They had sorted through and modified some activities once the NPO-led program ended, continuing with the activities thereafter with little change and several activity patterns: “continuity with modification” for cases of Nutrition Education and Peer Counseling, “relatively high continuity without modification” in Growth Monitoring, and “total discontinuation” in Cooking Demonstration. Such activity patterns may reflect the proactive ingenuity of CHWs, which can be a factor to promote program sustainability (2, 4, 22, 23), rather than program deterioration.

While more than half of the caregivers had participated in CHW activities, their attendance rates decreased over time, which contrasts with CHW activity continuation. If the decreased attendance had resulted from diminished local acceptance (local needs or awareness of CHW activities) (28), then the contrast might reflect CHW value judgment, i.e., which activities to continue, regardless of local acceptance. However, given that caregivers frequently mentioned CHW activities in the FGDs, the decreased attendance might instead reflect a change in CHW roles. Presumably, after acquiring sufficient knowledge about IYCF and child health, caregivers more readily conveyed the necessary information in casual conversations, which may have naturally discouraged caregiver attendance and affected CHW activities.

How the CHW valued and modified their activities, the local needs or awareness, or activities attended by the individual caregivers were unfortunately not investigated. The lack of this important information considerably limits any further discussion about the underlying reasons for CHW activity continuation. Other limitations include recall bias and social desirability bias in the questionnaire responses and FGDs. Considering the irregularity in activity instructions (i.e., Growth Monitoring), training opportunities, and activity targets, the amount of time elapsed from completion of the NPO-led program may also indicate differences in program quality or in the villages themselves, rather than serving as a simple time passage. In addition, changes in activity frequency, and activity quality were not analyzed. The program should be evaluated further according to recipient level health outcomes (i.e., improvement in child nutrition) (2).

## Conclusion

Even with these limitations, we still believe the CHW activity continuation observed in Thegon Township is worth presenting, as it may have been due to the proactive engagement of CHWs in the absence of supervision, and was potentially unlinked to the level of local acceptance, suggesting the importance of CHW autonomy (23) in their activity continuation. Future studies should focus on CHW autonomy.

## Data Availability Statement

The data analyzed in this study was obtained from Save the Children Japan. Requests to access the datasets should be directed to Taeko Oguma, t-oguma@jichi.ac.jp.

## Ethics Statement

This study was approved by the Ethics Committee of the Faculty of Medicine of the University of Tokyo (No. 110617). All the participants gave consent in the follow-up survey, also the NPO offered the participants an opportunity to opt-out before providing the anonymized data to the University of Tokyo through a social networking service (https://www.facebook.com/Thegon-child-nutrition-2007488195945235/ ^*^with limited accessible areas). The NPO also obtained ethical approval from the Ministry of Health and Sports, the Republic of the Union of Myanmar (Letter No. 116/Ethics 2015).

## Author Contributions

TO analyzed the data and wrote the initial draft. EW and TM critically reviewed and revised the manuscript. YF contributed to data interpretation and editing. All authors approved the final version.

## Conflict of Interest

TO participated in the NPO survey as a paid consultant in 2014. YF had worked as a paid employee during and after the NPO activity and the follow-up survey. The remaining authors declare that the research was conducted in the absence of any commercial or financial relationships that could be construed as a potential conflict of interest.
